# Measurement of stiffness in patients with rheumatoid arthritis in low disease activity or remission: a systematic review

**DOI:** 10.1186/1471-2474-15-28

**Published:** 2014-01-29

**Authors:** Lilian HD van Tuyl, Willem F Lems, Maarten Boers

**Affiliations:** 1 Department of Rheumatology, VU University Medical Center, PO Box 7057, 1007 MB Amsterdam, The Netherlands; 2 Department of Epidemiology and Biostatistics, VU University Medical Center, PO Box 7057, 1007 MB Amsterdam, The Netherlands

**Keywords:** Rheumatoid arthritis, Remission, Morning stiffness, Outcome assessment, Systematic review

## Abstract

**Background:**

Recent qualitative research has shown that stiffness is an important symptom for patients to identify remission. However, it is unclear how to measure stiffness in low disease activity. This systematic review aims to summarise the existing literature on validity of patient reported outcomes to measure stiffness in RA low disease activity states, to aid the choice for a measurement instrument.

**Methods:**

An extensive pubmed-search was undertaken, identifying measurement instruments for patient perceived stiffness used in low disease activity. Eligible studies reported on 1) stiffness as an outcome in relation to other core set measures, 2) development of a patient reported tool to measure stiffness, or 3) comparison of two different tools to measure aspects of stiffness, all in low disease activity.

**Results:**

Of 788 titles, only two studies report on validity of stiffness measures within low disease activity. Morning stiffness (MS) is reported in 44 to 80% of patients in low disease activity. A difference of 40 to 60 minutes in duration until maximum improvement is observed between active and inactive patients. Severity of MS might discriminate better between high and low disease activity compared to measurement of duration of MS.

**Conclusions:**

There is insufficient data on measurement of stiffness in the spectrum of low disease activity or remission.

## Background

Patients with rheumatoid arthritis (RA) often experience stiffness of joints, especially in the morning or after prolonged rest. Morning stiffness (MS) in RA can be attributed to the disrupted circadian rhythm of pro-inflammatory cytokine release in RA [1]. As a consequence, symptoms that follow the circadian rhythm like joint stiffness and pain are most severe in the early mornings [2].

Duration of MS was part of the American classification criteria for RA [3]. However, it was excluded from the recent update, as it was felt the instruments to measure it yielded data of insufficient reliability to include stiffness in the classification criteria [4-9]. Still, impaired morning function as a result of MS has considerable impact on the patients’ life. Severe stiffness in RA has been identified as the most important predictor of early retirement, apart from baseline working status [10]. A qualitative study showed significant impact of impaired morning function on quality of life, resulting in frustration and distress [11]. In addition, a strong inverse correlation between age and MS has been identified, with younger patients assessing their disease as more severe [12], pointing towards a higher burden of MS for younger patients compared to elderly patients. A qualitative study by Lineker et al. developed a patient-centered definition of MS in RA: 'slowness or difficulty moving the joints when getting out of bed or after staying in one position too long, which involves both sides of the body and gets better with movement’ [13].

Clearly, stiffness, either in the morning or during the day, is an important aspect of RA disease activity.

However, not much is known about the role of stiffness in low disease activity or remission. MS was part of the original ACR remission criteria [14], but is not a component of the new remission criteria for RA [15]. However, a recent qualitative study into the patients’ perspective on remission in RA showed that stiffness is an important aspect of the disease that needs to be reduced before one would feel to be in remission [16].

To investigate stiffness in low disease activity states, a valid measurement instrument is required. *Cutolo* recently published a bibliographic study of current assessment of morning stiffness, pain and function in RA [17]. Whilst a good overview of the current assessment and reporting of stiffness, this study did not report on performance of instruments in low disease activity.

With the current systematic review, we aim to summarise the existing literature on validity of patient reported outcomes to measure stiffness in RA low disease activity or remission, to aid the choice for a measurement instrument.

## Methods

An extensive literature search identified existing, feasible measurement instruments or subscales of existing instruments for patient perceived stiffness, developed or used within RA research. Identified studies were manually reviewed for evidence on performance of instruments in low disease activity states.

### Search strategy

We conducted a systematic literature search of Pubmed on 20-11-2012 to identify studies presenting patient reported measurement instruments that assess stiffness. No limitations for language or year of publication were used. The search was composed of three groups of search terms. The first group described the construct to be measured, ie stiffness. The second group described the disease, ie rheumatoid arthritis; the third group described the type of instrument, ie patient reported measurement instrument. These three groups were combined with a sensitive, validated filter that was specifically designed to identify studies on measurement properties in PubMed [18]. Finally, an exclusion filter was added, to remove non-relevant studies such as case reports, letters etc. The search strategy was composed by one author (LvT) in consultation with a medical information specialist (Table 1). See Additional file 1 for the detailed search strategy.

**Table 1:** Search strategy in PubMed

**Search**	**Topic**	**Result (hits)**
**1. Construct**	**Stiffness**	
Key words	stiffn*	32236
**2. Population**	**Adults with rheumatoid arthritis**	
Mesh	((“Arthritis, Rheumatoid”[Mesh:NoExp] OR “Caplan’s Syndrome”[Mesh]) OR “Felty’s Syndrome”[Mesh]) OR “Rheumatoid Nodule”[Mesh])	77.890
	OR	
Key words	(Arthriti*[tiab] OR rheuma*[tiab] OR caplan[tiab] OR felty[tiab])	172.447
		188.044
**3. Instrument**	**Patient reported outcome measures or instruments i.e. questionnaires, scales, subscales, indexes**	
Key words	(Questionnaire* OR scale OR instrument OR tool OR diary OR assessment OR self-report OR measure* OR PRO OR PROM)	3.428.574
**4. Sensitive filter **[18]	**1 + 2 + 3 AND sensitive filter**	
**5. exclusion filter **[18]	**4 NOT exclusion filter**	

*This symbol is used to search for all terms that start with this word.

Numbers 1 to 5 represent the different search blocks.

### Selection process and inclusion criteria

The selection of abstracts and full-text articles was done in four phases; first, one author (LvT) screened all titles and abstracts and selected titles that reported on either stiffness or function, on measurement of patient reported outcomes (PRO) or the development or validation of PRO instruments. Secondly, two authors (LvT and MB) independently from each other evaluated the remaining abstracts of the possibly relevant studies by specifically selecting on populations with RA and reporting of measurement properties of stiffness.

Thirdly, potentially relevant full articles were retrieved and independently evaluated by the above authors. Articles were selected when they reported on stiffness in one of the following ways:

1. stiffness as an outcome in relation to other core set disease activity measures

2. the development of a patient reported tool to measure stiffness

3. a comparison of two or more different tools to measure aspects of stiffness

The references of all studies included in the third phase, as well as reviews on measures of stiffness were checked for eligible studies.

**Figure 1:**
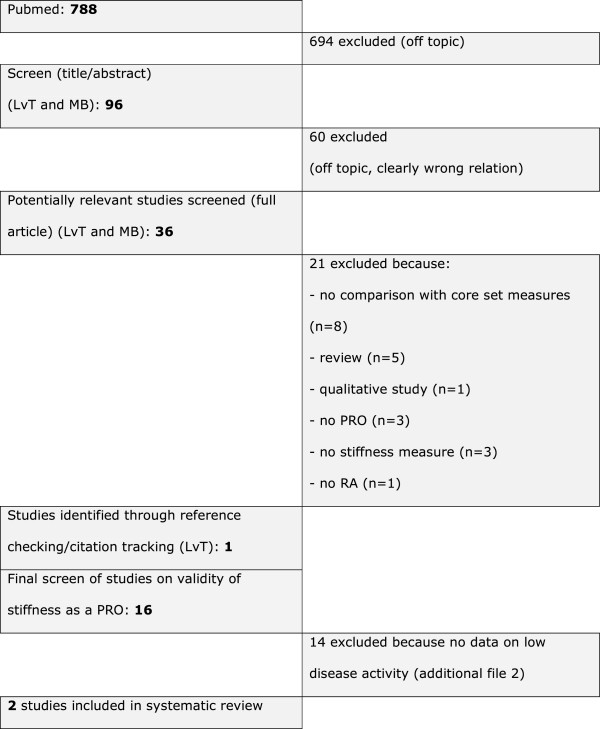
Flowchart of selection process.

Finally, the remaining articles, all reporting on aspects of validity of stiffness measures, were searched for information on performance in low disease activity or remission states by one author (LvT). See flowchart in Figure 1.

Data was extracted and summarised by one author (LvT) and reviewed by a second author (MB).

## Results

Of 788 titles, only 2 studies provide data on stiffness in low disease activity and are included in this review. Measurements are restricted to stiffness in the morning and include measures of duration (both studies) and severity (one study). Another 14 studies examined validity of stiffness as a PRO in RA disease activity in general (Additional file 2) [6,10,19-30]. The two studies in low disease activity included in this review are summarized below.

**Hazes et al. (1993)** investigated different measures of MS in 78 RA patients of median 60 years of age and a median disease duration of 9 years [31]. Two measures of MS severity and three measures of MS duration were compared (Table 2). Patients were grouped as 'active’ and 'inactive’ based on the physicians subjective impression of active joint inflammation.

**Table 2:** Identified measures of stiffness in rheumatoid arthritis low disease activity

**First author + yr of publ**	**Instrument and phrasing**	**n**
Hazes, 1993 [31]	1) Severity: 10 cm VAS 'no’ to 'very severe’	78
	2) Severity: NRS from 0 'no’ to 10 'very severe’.	
	3) Duration of MS (minutes):	
	1) *'how long does you MS last until it begins to improve?’*	
	*2) 'how long does your MS last until maximum improvement occurs?’*	
	*3) 'how long does it take you to get going properly?’*	
Khan, 2009 [32]	Duration of MS (minutes) in time from waking to time of max improvement in last week, in 4 categories:	5439
	none, mild (1–30), moderate (31–60) and severe (>60)	

MS was reported by 89% of patients in active and 81% in inactive disease. The duration of MS between the two groups was similar, with no significant differences between the groups, pointing to a lack of discriminative power of the three measures of MS duration. However, patients with active disease reported more severe MS than patients with inactive disease as measured by both severity scales, reflecting better discriminative properties for measures of severity vs measures of duration.

The sensitivity and specificity of the visual analogue scale (VAS) to distinguish between active and inactive disease was 85% and 44% respectively at a cut off of VAS > 2. In other words, with a MS VAS score of ≤2, there is a 44% chance to classify a patient correctly as inactive.

**Khan et al. (2009**) [32] investigated the utility of MS in assessing RA disease activity on data of the QUEST-RA database. A total of 5439 RA patients from 24 different countries and a mean disease duration of 11 years reported on MS duration from time of waking to time of maximal improvement. MS was studied separately for 4 different disease activity states classified according to DAS28 cut-offs for remission (<2.6), low (2.6 to ≤3.2), moderate (3.2 < to ≤ 5.1), and high (>5.1). In addition, the role of MS in Routine Assessment of Patient Index Data 3 (RAPID3, composed of the core set PROs pain, global health and physical functioning) to predict disease activity according to the DAS28 was explored.

MS duration was significantly different for each disease activity state, with the smallest difference (9 minutes) between low disease activity and remission. Although the duration of MS increased with disease activity, even patients whose disease seemed to be under control reported considerable duration of morning stiffness: of the 1594 patients with a DAS28 ≤ 3.2, 16% experienced morning stiffness of more than half an hour, of which 46% with a duration of more than one hour. Positive likelihood ratios of having active disease for different MS duration categories rose from 0.35 (0 min) to 4 (>60 min).

The accuracy of MS to differentiate between active and inactive disease (DAS28 cut off 3.2) was moderate, represented by the area under the receiver operating curve: 0.74 (0.72-0.75).

The relationship between RAPID3 and DAS28 was significantly different for each MS duration category. Interestingly, MS duration was especially valuable for RAPID3 scores representing low disease activity, where patients with low RAPID3 scores but a DAS28 > 3.2 were identified by presence of MS.

## Discussion

This review identified 2 studies that investigated measures of stiffness in RA low disease activity states, both focussed on *morning* stiffness. There are large differences between the 2 studies in patient characteristics as well as in collection of outcome measures, making it difficult to draw overarching conclusions. Most importantly, this review highlights the lack of scientific data on the performance of stiffness measures in low disease activity states.

Both studies included in this review show that despite suppressed disease activity, morning stiffness is still present; in the Hazes study, 89 vs 80% and in the Khan study 79 vs 44% of active vs inactive patients report MS.

The study of Hazes et al. concludes that severity of MS measured with a VAS or NRS scale can discriminate between active and inactive disease, in contrast to measures of duration. On the other hand, Khan et al., show that the same measure of duration (measured in minutes from time of awaking to time of maximal improvement), in a much larger sample of RA patients, does show moderate discrimination between active and inactive RA. As the two studies differ in several ways, conflicting conclusions are not surprising. However, when looking closely, both studies show a similar difference in MS duration between active and inactive RA patients, with a difference of 60 minutes in the Hazes-study and 40 minutes in the Khan-study. This means that the most likely explanation for the difference in observations is the statistical power of the studies to detect differences in duration of MS between active and inactive patients, with the Hazes-study including 78 patients and the Khan-study 5439 patients; however, differences can also be attributed to differences in cut-off points for active and inactive RA (physician based vs DAS28 based); or to a change in patients perception of stiffness over the years in comparison to other RA symptoms and outcomes, as the Hazes study was published in 1993 and the Khan study in 2009. More research is needed to identify the best instrument to measure MS in low disease activity; not only focused on discrimination between active and inactive RA, but also on construct validity (whether low MS scores correlate with low scores on other disease activity measures) and sensitivity to pick up changes between low disease activity and remission states.

A limitation of this review of the literature is the decision not to document evidence of response of stiffness measures to therapy. As many studies do not mention stiffness in title, abstract or keywords this would have required a full-text review of almost every RA intervention study. Therefore, a specific search strategy was adopted in combination with a sensitive methodological PubMed search filter that is specially designed and validated for finding studies on measurement properties of measurement instruments [18].

Several studies provide evidence for validity of both duration as well as severity scales to measure stiffness, as both generally show good correlation with core set measures (especially pain and function) [19-22,27,28,30]. However, problems arise when these measures are used to discriminate RA from non-inflammatory conditions or osteoarthritis [12,24,26,31]. Yet, this is only relevant when it is used as a diagnostic criterion, which justifies the removal of stiffness as a component of the ACR classification criteria.

In general, studies comparing measures of duration with severity of stiffness show better measurement properties for severity in terms of sensitivity to change over time, responsiveness to therapy and reliability [10,29,31]. This is confirmed by the Hazes study included in this review, that shows discrimination between active and inactive disease of severity scales but not for measurement of duration of MS.

Qualitative studies investigating the concept of stiffness in RA point to the strong connection between aspects of stiffness and aspects of pain and physical functioning [25,31]. This is confirmed by the Khan study, where MS correlates better with physical function, pain and global health than with joint counts or ESR. However, no specific measurement instruments have been developed from these qualitative explorations on the concept of stiffness. The observation that MS duration adds important information to predicting disease activity specifically at low RAPID3 scores is highly interesting, pointing to a possible distinguishing role for MS in classification of remission. To evaluate the contribution of MS to the newly developed ACR/EULAR remission criteria, new prospective studies on the performance of different MS measures in the spectrum of low disease activity are needed.

## Conclusion

In conclusion: while patients in low disease activity frequently report stiffness, the tools to measure stiffness are of unknown validity due to a lack of data on performance of patient reported measures of stiffness in RA low disease activity or remission.

## Competing interests

The authors have no competing interest concerning this study.

## Authors’ contributions

All authors (LH, WF, MB) have made substantial contributions to conception and design, acquisition of data, analysis and interpretation of data; have been involved in drafting the manuscript and have given final approval of the version to be published.

## Pre-publication history

The pre-publication history for this paper can be accessed here:

http://www.biomedcentral.com/1471-2474/15/28/prepub

## Supplementary Material

Additional file 1 Detailed search strategy. Click here for file

Additional file 2 Published articles on instruments or subscales of instruments to measure stiffness. Click here for file
