# Supplementary material for: Measurement of stiffness in patients with rheumatoid arthritis in low disease activity or remission: a systematic review
Source: BMC Musculoskelet Disord. 2014 Jan 29;15:28. doi: 10.1186/1471-2474-15-28 (PMC3914735; doi:10.1186/1471-2474-15-28)
Supplement: Additional file 1 — Detailed search strategy. [file 1471-2474-15-28-S1.doc]

**Additional file 1 Detailed search strategy**

| **1. construct: stiffness>stiffn*** |
| --- |
| stiffneck[All Fields] OR stiffnes[All Fields] OR stiffness[All Fields] OR stiffness/abnormal[All Fields] OR stiffness/arthropathy/contractures[All Fields] OR stiffness/compliance[All Fields] OR stiffness/concentration[All Fields] OR stiffness/cramps[All Fields] OR stiffness/deflection[All Fields] OR stiffness/density[All Fields] OR stiffness/effection[All Fields] OR stiffness/elasticity[All Fields] OR stiffness/endothelial[All Fields] OR stiffness/fibrosis[All Fields] OR stiffness/flexibility[All Fields] OR stiffness/force[All Fields] OR stiffness/free[All Fields] OR stiffness/function[All Fields] OR stiffness/impedance[All Fields] OR stiffness/lameness[All Fields] OR stiffness/lumbago[All Fields] OR stiffness/mass[All Fields] OR stiffness/modules[All Fields] OR stiffness/pain[All Fields] OR stiffness/physical[All Fields] OR stiffness/pressure[All Fields] OR stiffness/proximal[All Fields] OR stiffness/quantitative[All Fields] OR stiffness/qui[All Fields] OR stiffness/recoil[All Fields] OR stiffness/rigidity[All Fields] OR stiffness/soreness[All Fields] OR stiffness/spasm[All Fields] OR stiffness/spasticity[All Fields] OR stiffness/spleen[All Fields] OR stiffness/strength[All Fields] OR stiffness/strengths[All Fields] OR stiffness/suppleness[All Fields] OR stiffness/tension[All Fields] OR stiffness/thickness[All Fields] OR stiffness/tiredness[All Fields] OR stiffness/viscoelasticity[All Fields] OR stiffness/wave[All Fields] OR stiffness/weakness[All Fields] OR stiffness'[All Fields] OR stiffnesses[All Fields] OR stiffnessess[All Fields] OR stiffnessindexin[All Fields] OR stiffnessparameters[All Fields] OR stiffnesss[All Fields] OR stiffnessxvertical[All Fields] OR stiffnest[All Fields] |
| **2. population: adults with rheumatoid arthritis** |
| (("Arthritis, Rheumatoid"[Mesh:noexp] OR "Felty's Syndrome"[Mesh]) OR "Rheumatoid Nodule"[Mesh]) OR ((arthritiaand[tiab] OR arthritic[tiab] OR arthritic/contralateral[tiab] OR arthritic/rheumatoid[tiab] OR arthritic'[tiab] OR arthritica[tiab] OR arthritically[tiab] OR arthritici[tiab] OR arthriticrats[tiab] OR arthritics[tiab] OR arthriticum[tiab] OR arthritide[tiab] OR arthritides[tiab] OR arthritides/spondyloarthropathies[tiab] OR arthritides/synovitides[tiab] OR arthritidies[tiab] OR arthritidis[tiab] OR arthritidities[tiab] OR arthritidy[tiab] OR arthrities[tiab] OR arthritigen[tiab] OR arthritigenic[tiab] OR arthritigens[tiab] OR arthritiis[tiab] OR arthritin[tiab] OR arthritin'[tiab] OR arthritiogenic[tiab] OR arthritis[tiab] OR arthritis/adult[tiab] OR arthritis/ankylosis[tiab] OR arthritis/arthalgia[tiab] OR arthritis/arthralgia[tiab] OR arthritis/arthralgias[tiab] OR arthritis/arthropathy[tiab] OR arthritis/arthrosis[tiab] OR arthritis/arthrosis/rheumatic[tiab] OR arthritis/back[tiab] OR arthritis/backache[tiab] OR arthritis/bursitis[tiab] OR arthritis/ccl[tiab] OR arthritis/cellulitis[tiab] OR arthritis/chronic[tiab] OR arthritis/cjs[tiab] OR arthritis/connective[tiab] OR arthritis/deformity[tiab] OR arthritis/dermatitis[tiab] OR arthritis/diabetes[tiab] OR arthritis/disseminated[tiab] OR arthritis/dysfunction[tiab] OR arthritis/early[tiab] OR arthritis/encephalitis[tiab] OR arthritis/enthesitis[tiab] OR arthritis/fibrosis[tiab] OR arthritis/gout[tiab] OR arthritis/high[tiab] OR arthritis/infectious[tiab] OR arthritis/inflammatory[tiab] OR arthritis/jaccoud's[tiab] OR arthritis/joint[tiab] OR arthritis/juvenile[tiab] OR arthritis/lameness[tiab] OR arthritis/low[tiab] OR arthritis/lupus[tiab] OR arthritis/mobility[tiab] OR arthritis/musculoskeletal[tiab] OR arthritis/myositis[tiab] OR arthritis/osteoarthritis[tiab] OR arthritis/osteomyelitis[tiab] OR arthritis/osteoporosis[tiab] OR arthritis/other[tiab] OR arthritis/pain[tiab] OR arthritis/periarthritis[tiab] OR arthritis/placebo[tiab] OR arthritis/polyarthritis[tiab] OR arthritis/polymyalgia[tiab] OR arthritis/polymyositis[tiab] OR arthritis/prosthetic[tiab] OR arthritis/psoriasis[tiab] OR arthritis/pyomyositis[tiab] OR arthritis/ra[tiab] OR arthritis/reiter's[tiab] OR arthritis/rheumatic[tiab] OR arthritis/rheumatism[tiab] OR arthritis/rheumatoid[tiab] OR arthritis/rheumatology[tiab] OR arthritis/scid[tiab] OR arthritis/sjogren's[tiab] OR arthritis/spondylitis[tiab] OR arthritis/spondyloarthropathy[tiab] OR arthritis/ss[tiab] OR arthritis/statin[tiab] OR arthritis/synovitis[tiab] OR arthritis/systemic[tiab] OR arthritis/tendonitis[tiab] OR arthritis/tenosynovitis[tiab] OR arthritis/tophaceous[tiab] OR arthritis/undifferentiated[tiab] OR arthritis/urethritis/conjunctivitis[tiab] OR arthritis/vasculitis[tiab] OR arthritis/vasculitis/autoimmune/collagen[tiab] OR arthritis'[tiab] OR arthritis''[tiab] OR arthritis's[tiab] OR arthritisasymmetrical[tiab] OR arthritisb[tiab] OR arthritisbut[tiab] OR arthritisdoctors[tiab] OR arthritise[tiab] OR arthritises[tiab] OR arthritisiare[tiab] OR arthritisis[tiab] OR arthritislike[tiab] OR arthritism[tiab] OR arthritismus[tiab] OR arthritisp6[tiab] OR arthritispathology[tiab] OR arthritisprone[tiab] OR arthritisrelated[tiab] OR arthritisstudies[tiab] OR arthritist[tiab] OR arthritiswith[tiab] OR arthrititis[tiab] OR arthrititis/asiatica[tiab] OR arthritits[tiab] OR arthritix[tiab]) OR (rheuma[tiab] OR rheuma'[tiab] OR rheumabad[tiab] OR rheumabene[tiab] OR rheumacenters[tiab] OR rheumacheck[tiab] OR rheumacoach[tiab] OR rheumacoach'[tiab] OR rheumadat[tiab] OR rheumaderm[tiab] OR rheumaeinheit[tiab] OR rheumafactor[tiab] OR rheumafactors[tiab] OR rheumafaktor[tiab] OR rheumafoot[tiab] OR rheumaforschg[tiab] OR rheumaforschung[tiab] OR rheumaforschungsprogramme[tiab] OR rheumaforschungszentrum[tiab] OR rheumagen[tiab] OR rheumaid[tiab] OR rheumaitc[tiab] OR rheumaitism[tiab] OR rheumajecta[tiab] OR rheumaklinik[tiab] OR rheumakutin[tiab] OR rheumalatex[tiab] OR rheumaliga[tiab] OR rheumalysin[tiab] OR rheumanet[tiab] OR rheumanol[tiab] OR rheumaoid[tiab] OR rheumaorthopadie[tiab] OR rheumaorthopaedic[tiab] OR rheumaorthopaedics[tiab] OR rheumapathology[tiab] OR rheumapax[tiab] OR rheumarthopaedics[tiab] OR rheumarthritis[tiab] OR rheumarthropatic[tiab] OR rheumasan[tiab] OR rheumaserology[tiab] OR rheumasit[tiab] OR rheumastrip[tiab] OR rheumasurgeon[tiab] OR rheumasurgeons[tiab] OR rheumasurgery[tiab] OR rheumasurgical[tiab] OR rheumat[tiab] OR rheumatalgia[tiab] OR rheumatalgic[tiab] OR rheumatalogic[tiab] OR rheumateam[tiab] OR rheumateid[tiab] OR rheumatest[tiab] OR rheumatherapy[tiab] OR rheumathoid[tiab] OR rheumathologic[tiab] OR rheumathology[tiab] OR rheumati[tiab] OR rheumatic[tiab] OR rheumatic/autoimmune[tiab] OR rheumatic/congenital[tiab] OR rheumatic/connective[tiab] OR rheumatic/inflammatory[tiab] OR rheumatic/orthopaedic[tiab] OR rheumatic/overlap[tiab] OR rheumatic'[tiab] OR rheumatica[tiab] OR rheumatica/arteritis[tiab] OR rheumatica/gca[tiab] OR rheumatica/giant[tiab] OR rheumatica/temporal[tiab] OR rheumatica'[tiab] OR rheumaticae[tiab] OR rheumatical[tiab] OR rheumatically[tiab] OR rheumatiche[tiab] OR rheumatickies[tiab] OR rheumatics[tiab] OR rheumaticum[tiab] OR rheumaticus[tiab] OR rheumatid[tiab] OR rheumatie[tiab] OR rheumatif[tiab] OR rheumatim[tiab] OR rheumatims[tiab] OR rheumatio[tiab] OR rheumatiod[tiab] OR rheumatioid[tiab] OR rheumation[tiab] OR rheumatis[tiab] OR rheumatisant[tiab] OR rheumatische[tiab] OR rheumatism[tiab] OR rheumatism/american[tiab] OR rheumatism/arthritis[tiab] OR rheumatism/arthritis/arthrosis[tiab] OR rheumatism/eular[tiab] OR rheumatism/european[tiab] OR rheumatism/myalgia[tiab] OR rheumatism/osteoarthritis[tiab] OR rheumatism/paediatric[tiab] OR rheumatism/pediatric[tiab] OR rheumatism/pres[tiab] OR rheumatism'[tiab] OR rheumatismal[tiab] OR rheumatismes[tiab] OR rheumatismm[tiab] OR rheumatisms[tiab] OR rheumatismus[tiab] OR rheumatitis[tiab] OR rheumato[tiab] OR rheumatoarthritic[tiab] OR rheumatoarthrological[tiab] OR rheumatobates[tiab] OR rheumatod[tiab] OR rheumatodi[tiab] OR rheumatoed[tiab] OR rheumatogenetic[tiab] OR rheumatogenic[tiab] OR rheumatogenicity[tiab] OR rheumatogenous[tiab] OR rheumatogical[tiab] OR rheumatoic[tiab] OR rheumatoid[tiab] OR rheumatoid/idiopathic[tiab] OR rheumatoid/inflammatory[tiab] OR rheumatoid/lupus[tiab] OR rheumatoid/osteoarthritis/connective[tiab] OR rheumatoid'[tiab] OR rheumatoidal[tiab] OR rheumatoidarthritis[tiab] OR rheumatoidd[tiab] OR rheumatoide[tiab] OR rheumatoidea[tiab] OR rheumatoides[tiab] OR rheumatoidfactor[tiab] OR rheumatoidjoint[tiab] OR rheumatoidlike[tiab] OR rheumatoidpolyarthritis[tiab] OR rheumatoids[tiab] OR rheumatoidtero[tiab] OR rheumatois[tiab] OR rheumatoism[tiab] OR rheumatol[tiab] OR rheumatold[tiab] OR rheumatolgy[tiab] OR rheumatolog[tiab] OR rheumatologial[tiab] OR rheumatologic[tiab] OR rheumatologic/autoimmune[tiab] OR rheumatologic/inflammatory[tiab] OR rheumatologic/neurologic[tiab] OR rheumatologic/orthopedic[tiab] OR rheumatologic/orthopedic/psychiatric[tiab] OR rheumatologic/renal[tiab] OR rheumatological[tiab] OR rheumatological/autoimmune[tiab] OR rheumatological/neurological[tiab] OR rheumatological'[tiab] OR rheumatologically[tiab] OR rheumatologie[tiab] OR rheumatologisch[tiab] OR rheumatologische[tiab] OR rheumatologist[tiab] OR rheumatologist/clinical[tiab] OR rheumatologist/epidemiologist[tiab] OR rheumatologist/internist[tiab] OR rheumatologist/month[tiab] OR rheumatologist'[tiab] OR rheumatologist's[tiab] OR rheumatologists[tiab] OR rheumatologists/clinical[tiab] OR rheumatologists/immunologists[tiab] OR rheumatologists/internists[tiab] OR rheumatologists/internists/laboratory[tiab] OR rheumatologists/osteologists[tiab] OR rheumatologists/pain[tiab] OR rheumatologists/population[tiab] OR rheumatologists'[tiab] OR rheumatologoy[tiab] OR rheumatology[tiab] OR rheumatology/allergy[tiab] OR rheumatology/arthritis[tiab] OR rheumatology/association[tiab] OR rheumatology/british[tiab] OR rheumatology/dermatology[tiab] OR rheumatology/eular[tiab] OR rheumatology/european[tiab] OR rheumatology/general[tiab] OR rheumatology/immunology[tiab] OR rheumatology/orthopedic[tiab] OR rheumatology/osteoarthritis[tiab] OR rheumatology/osteoporosis[tiab] OR rheumatology/pain[tiab] OR rheumatology/physical[tiab] OR rheumatology/rheumatic[tiab] OR rheumatology/stpr[tiab] OR rheumatology/systemic[tiab] OR rheumatology'[tiab] OR rheumatology's[tiab] OR rheumatologyas[tiab] OR rheumatologyworking[tiab] OR rheumatolological[tiab] OR rheumatoloy[tiab] OR rheumaton[tiab] OR rheumatontest[tiab] OR rheumatoorthopedic[tiab] OR rheumatoorthopedics[tiab] OR rheumatorid[tiab] OR rheumatoserology[tiab] OR rheumatosis[tiab] OR rheumatosurgery[tiab] OR rheumatotests[tiab] OR rheumatrex[tiab] OR rheumaturic[tiab] OR rheumax[tiab] OR rheumazahl[tiab] OR rheumazentrum[tiab] OR rheumazin[tiab]) OR caplan[tiab] OR felty[tiab]) |
| **3. Instrument: PRO>** (questionnaire* OR scale OR instrument OR tool OR diary OR assessment OR self-report OR measure* OR PRO OR PROM) |
| (questionnaire[All Fields] OR questionnaire/algorithm[All Fields] OR questionnaire/beck[All Fields] OR questionnaire/chart[All Fields] OR questionnaire/checklist[All Fields] OR questionnaire/childhood[All Fields] OR questionnaire/consent[All Fields] OR questionnaire/diary[All Fields] OR questionnaire/examination[All Fields] OR questionnaire/form[All Fields] OR questionnaire/forward[All Fields] OR questionnaire/general[All Fields] OR questionnaire/german[All Fields] OR questionnaire/haq[All Fields] OR questionnaire/health[All Fields] OR questionnaire/index[All Fields] OR questionnaire/inquiry[All Fields] OR questionnaire/interview[All Fields] OR questionnaire/itaq[All Fields] OR questionnaire/lateral[All Fields] OR questionnaire/medical[All Fields] OR questionnaire/module[All Fields] OR questionnaire/negative[All Fields] OR questionnaire/pain[All Fields] OR questionnaire/patients[All Fields] OR questionnaire/physician[All Fields] OR questionnaire/record[All Fields] OR questionnaire/routine[All Fields] OR questionnaire/scale[All Fields] OR questionnaire/schedule[All Fields] OR questionnaire/school[All Fields] OR questionnaire/self[All Fields] OR questionnaire/sense[All Fields] OR questionnaire/sketch[All Fields] OR questionnaire/subscale[All Fields] OR questionnaire/surgery[All Fields] OR questionnaire/survey[All Fields] OR questionnaire/telephone[All Fields] OR questionnaire/tool[All Fields] OR questionnaire/tool/instrument[All Fields] OR questionnaire/urogenital[All Fields] OR questionnaire/validation[All Fields] OR questionnaire/western[All Fields] OR questionnaire'[All Fields] OR questionnaire'07[All Fields] OR questionnaire's[All Fields] OR questionnaire1[All Fields] OR questionnaire12[All Fields] OR questionnaire25[All Fields] OR questionnaire30[All Fields] OR questionnaireand[All Fields] OR questionnairebased[All Fields] OR questionnairecopyright[All Fields] OR questionnaired[All Fields] OR questionnairel02[All Fields] OR questionnairenurse[All Fields] OR questionnairepf[All Fields] OR questionnaires[All Fields] OR questionnaires/25[All Fields] OR questionnaires/blood[All Fields] OR questionnaires/checklists[All Fields] OR questionnaires/classification[All Fields] OR questionnaires/economics[All Fields] OR questionnaires/examinations[All Fields] OR questionnaires/interviews[All Fields] OR questionnaires/methods[All Fields] OR questionnaires/modules[All Fields] OR questionnaires/personal[All Fields] OR questionnaires/standards[All Fields] OR questionnaires/subject[All Fields] OR questionnaires/subscales[All Fields] OR questionnaires/tasks[All Fields] OR questionnaires/telephone[All Fields] OR questionnaires/tests[All Fields] OR questionnaires/trends[All Fields] OR questionnaires/utilization[All Fields] OR questionnaires/veterinary[All Fields] OR questionnaires'[All Fields] OR questionnaires''[All Fields] OR questionnairescan[All Fields] OR questionnairestrial[All Fields] OR questionnairetrade[All Fields] OR questionnairev[All Fields] OR questionnairey[All Fields]) OR ("weights and measures"[MeSH Terms] OR ("weights"[All Fields] AND "measures"[All Fields]) OR "weights and measures"[All Fields] OR "scale"[All Fields]) OR instrument[All Fields] OR tool[All Fields] OR diary[All Fields] OR ("Assessment"[Journal] OR "assessment"[All Fields]) OR ("self report"[MeSH Terms] OR ("self"[All Fields] AND "report"[All Fields]) OR "self report"[All Fields]) OR (measure[All Fields] OR measure/15[All Fields] OR measure/analysis[All Fields] OR measure/barthel[All Fields] OR measure/behavior[All Fields] OR measure/cm[All Fields] OR measure/demographic[All Fields] OR measure/document[All Fields] OR measure/evaluation[All Fields] OR measure/function[All Fields] OR measure/functional[All Fields] OR measure/h[All Fields] OR measure/index[All Fields] OR measure/indicator[All Fields] OR measure/meter[All Fields] OR measure/monitor[All Fields] OR measure/observe[All Fields] OR measure/procedure[All Fields] OR measure/quantify[All Fields] OR measure/quantitate[All Fields] OR measure/s[All Fields] OR measure/split[All Fields] OR measure/sustain[All Fields] OR measure'[All Fields] OR measure's[All Fields] OR measure1[All Fields] OR measure2[All Fields] OR measure3[All Fields] OR measure4[All Fields] OR measurea[All Fields] OR measureable[All Fields] OR measureables[All Fields] OR measureably[All Fields] OR measureble[All Fields] OR measurechanges[All Fields] OR measured[All Fields] OR measured/1[All Fields] OR measured/1,580[All Fields] OR measured/2[All Fields] OR measured/206[All Fields] OR measured/824[All Fields] OR measured/actual[All Fields] OR measured/calculated[All Fields] OR measured/collected[All Fields] OR measured/counted[All Fields] OR measured/determined[All Fields] OR measured/documented[All Fields] OR measured/estimated[All Fields] OR measured/expected[All Fields] OR measured/given[All Fields] OR measured/imputed[All Fields] OR measured/inferred[All Fields] OR measured/interpolated[All Fields] OR measured/isd[All Fields] OR measured/modeled[All Fields] OR measured/mol[All Fields] OR measured/observed[All Fields] OR measured/pain[All Fields] OR measured/patient[All Fields] OR measured/performed[All Fields] OR measured/phr[All Fields] OR measured/predicted[All Fields] OR measured/quantified[All Fields] OR measured/reported[All Fields] OR measured/response[All Fields] OR measured/sigma[All Fields] OR measured/simulated[All Fields] OR measured/urine[All Fields] OR measured/v50[All Fields] OR measured'[All Fields] OR measured'characteristics[All Fields] OR measured2[All Fields] OR measuredaperture[All Fields] OR measuredat[All Fields] OR measuredby[All Fields] OR measureddrop[All Fields] OR measuredfluorometrically[All Fields] OR measuredhs[All Fields] OR measuredin[All Fields] OR measuredmore[All Fields] OR measuredmthe[All Fields] OR measuredp[All Fields] OR measuredu[All Fields] OR measuredweekly[All Fields] OR measuree[All Fields] OR measureed[All Fields] OR measureements[All Fields] OR measureent[All Fields] OR measurees[All Fields] OR measurefrom[All Fields] OR measureimprovement[All Fields] OR measureing[All Fields] OR measureiwe[All Fields] OR measureless[All Fields] OR measurels[All Fields] OR measuremed[All Fields] OR measuremehts[All Fields] OR measurememt[All Fields] OR measurememts[All Fields] OR measuremen[All Fields] OR measuremennt[All Fields] OR measuremenportion[All Fields] OR measuremens[All Fields] OR measuremenst[All Fields] OR measurement[All Fields] OR measurement/0[All Fields] OR measurement/acquisition[All Fields] OR measurement/analysis[All Fields] OR measurement/assessment[All Fields] OR measurement/assessments[All Fields] OR measurement/aus[All Fields] OR measurement/basic[All Fields] OR measurement/calculation[All Fields] OR measurement/control[All Fields] OR measurement/correction[All Fields] OR measurement/cranial[All Fields] OR measurement/data[All Fields] OR measurement/day[All Fields] OR measurement/definition[All Fields] OR measurement/documentation[All Fields] OR measurement/electrical[All Fields] OR measurement/electrocardiography[All Fields] OR measurement/estimation[All Fields] OR measurement/estrogen[All Fields] OR measurement/evaluation[All Fields] OR measurement/feedback[All Fields] OR measurement/findings[All Fields] OR measurement/haunch[All Fields] OR measurement/hypothesis[All Fields] OR measurement/improvement[All Fields] OR measurement/index[All Fields] OR measurement/intervention[All Fields] OR measurement/limits[All Fields] OR measurement/main[All Fields] OR measurement/management[All Fields] OR measurement/mg[All Fields] OR measurement/model[All Fields] OR measurement/monitoring[All Fields] OR measurement/numerical[All Fields] OR measurement/observer[All Fields] OR measurement/outcome[All Fields] OR measurement/p[All Fields] OR measurement/participants[All Fields] OR measurement/phonetography[All Fields] OR measurement/plaque[All Fields] OR measurement/pre[All Fields] OR measurement/prediction[All Fields] OR measurement/processing[All Fields] OR measurement/processing/telemetry[All Fields] OR measurement/renewal[All Fields] OR measurement/reporting[All Fields] OR measurement/s[All Fields] OR measurement/single[All Fields] OR measurement/standard[All Fields] OR measurement/subject[All Fields] OR measurement/systems[All Fields] OR measurement/time[All Fields] OR measurement/total[All Fields] OR measurement/transjugular[All Fields] OR measurement/verification[All Fields] OR measurement/year[All Fields] OR measurement'[All Fields] OR measurement's[All Fields] OR measuremental[All Fields] OR measurementd[All Fields] OR measurementexhaled[All Fields] OR measurementf[All Fields] OR measurementin[All Fields] OR measuremention[All Fields] OR measurementl[All Fields] OR measurementmanometry[All Fields] OR measurementof[All Fields] OR measurementpro[All Fields] OR measurementresearch[All Fields] OR measurements[All Fields] OR measurements/30mmhg[All Fields] OR measurements/4[All Fields] OR measurements/40mmhg[All Fields] OR measurements/analyte[All Fields] OR measurements/animal[All Fields] OR measurements/assay[All Fields] OR measurements/assessments[All Fields] OR measurements/calculations[All Fields] OR measurements/case[All Fields] OR measurements/child[All Fields] OR measurements/d[All Fields] OR measurements/data[All Fields] OR measurements/day[All Fields] OR measurements/day/person[All Fields] OR measurements/day/variable/subject[All Fields] OR measurements/device[All Fields] OR measurements/dexa[All Fields] OR measurements/diagnostic[All Fields] OR measurements/diaries[All Fields] OR measurements/entries[All Fields] OR measurements/eye[All Fields] OR measurements/findings[All Fields] OR measurements/flow[All Fields] OR measurements/four[All Fields] OR measurements/h[All Fields] OR measurements/hour[All Fields] OR measurements/hr[All Fields] OR measurements/indices[All Fields] OR measurements/interfacial[All Fields] OR measurements/intracanal[All Fields] OR measurements/inversion[All Fields] OR measurements/kidney[All Fields] OR measurements/main[All Fields] OR measurements/mean[All Fields] OR measurements/measurers[All Fields] OR measurements/method[All Fields] OR measurements/methods[All Fields] OR measurements/monitoring[All Fields] OR measurements/n[All Fields] OR measurements/number[All Fields] OR measurements/observations[All Fields] OR measurements/patient[All Fields] OR measurements/person[All Fields] OR measurements/plethysmography[All Fields] OR measurements/results[All Fields] OR measurements/results/conclusion[All Fields] OR measurements/s[All Fields] OR measurements/school[All Fields] OR measurements/sd[All Fields] OR measurements/serum[All Fields] OR measurements/standards[All Fields] OR measurements/statistical[All Fields] OR measurements/steer[All Fields] OR measurements/subject[All Fields] OR measurements/surrogates[All Fields] OR measurements/surveys[All Fields] OR measurements/tests[All Fields] OR measurements/three[All Fields] OR measurements/time[All Fields] OR measurements/tooth[All Fields] OR measurements/total[All Fields] OR measurements/week[All Fields] OR measurements/woman[All Fields] OR measurements/year[All Fields] OR measurements'[All Fields] OR measurements's[All Fields] OR measurements0[All Fields] OR measurements5[All Fields] OR measurementsa[All Fields] OR measurementscheme[All Fields] OR measurementsfor[All Fields] OR measurementsgave[All Fields] OR measurementsin[All Fields] OR measurementsindicate[All Fields] OR measurementsof[All Fields] OR measurementson[All Fields] OR measurementsreveal[All Fields] OR measurementswere[All Fields] OR measurementtime[All Fields] OR measurementts[All Fields] OR measurementusing[All Fields] OR measurementws[All Fields] OR measuremetns[All Fields] OR measuremets[All Fields] OR measuremeuts[All Fields] OR measuremnent[All Fields] OR measuremnents[All Fields] OR measuremnet[All Fields] OR measuremnt[All Fields] OR measuremnts[All Fields] OR measuremodel[All Fields] OR measuremouse[All Fields] OR measuremrnt[All Fields] OR measuremtn[All Fields] OR measuremtnes[All Fields] OR measuremtns[All Fields] OR measuremtnt[All Fields] OR measurend[All Fields] OR measurends[All Fields] OR measurenent[All Fields] OR measurenment[All Fields] OR measurenments[All Fields] OR measurent[All Fields] OR measurents[All Fields] OR measurenumber[All Fields] OR measureoutcomes[All Fields] OR measureoxy[All Fields] OR measurephantom[All Fields] OR measurepoint[All Fields] OR measurepoints[All Fields] OR measureprimary[All Fields] OR measurer[All Fields] OR measurer'[All Fields] OR measurer's[All Fields] OR measurerment[All Fields] OR measurerments[All Fields] OR measurers[All Fields] OR measurers/testers[All Fields] OR measurers'[All Fields] OR measures[All Fields] OR measures/alarms[All Fields] OR measures/analyses[All Fields] OR measures/analysis[All Fields] OR measures/batteries[All Fields] OR measures/courses[All Fields] OR measures/criteria[All Fields] OR measures/data[All Fields] OR measures/day[All Fields] OR measures/death[All Fields] OR measures/design[All Fields] OR measures/drugs[All Fields] OR measures/early[All Fields] OR measures/factor[All Fields] OR measures/h[All Fields] OR measures/history[All Fields] OR measures/indicators[All Fields] OR measures/indicators/contributors[All Fields] OR measures/indices[All Fields] OR measures/interventions[All Fields] OR measures/longitudinal[All Fields] OR measures/mass[All Fields] OR measures/methods[All Fields] OR measures/metric[All Fields] OR measures/metrics[All Fields] OR measures/outcomes[All Fields] OR measures/physical[All Fields] OR measures/physiotherapy[All Fields] OR measures/placebos[All Fields] OR measures/questionnaires[All Fields] OR measures/ratio[All Fields] OR measures/regulations[All Fields] OR measures/reporters[All Fields] OR measures/response[All Fields] OR measures/results[All Fields] OR measures/s[All Fields] OR measures/safety[All Fields] OR measures/scales[All Fields] OR measures/scores[All Fields] OR measures/screening[All Fields] OR measures/signals[All Fields] OR measures/skin[All Fields] OR measures/statistical[All Fields] OR measures/study[All Fields] OR measures/subject[All Fields] OR measures/subjects[All Fields] OR measures/surface[All Fields] OR measures/symbols[All Fields] OR measures/tasks[All Fields] OR measures/tests[All Fields] OR measures/tukey's[All Fields] OR measures/univariate[All Fields] OR measures/volume[All Fields] OR measures'[All Fields] OR measuresalzheimer's[All Fields] OR measuresanova[All Fields] OR measurescope[All Fields] OR measuresdescriptive[All Fields] OR measurese[All Fields] OR measureses[All Fields] OR measuresf[All Fields] OR measuresfatigue[All Fields] OR measuresfor[All Fields] OR measuresinterrater[All Fields] OR measuresl[All Fields] OR measuresm[All Fields] OR measuresments[All Fields] OR measuresnumber[All Fields] OR measurespectively[All Fields] OR measuresprimary[All Fields] OR measuresrates[All Fields] OR measuresresults[All Fields] OR measurestate[All Fields] OR measuresurement[All Fields] OR measurethe[All Fields] OR measuretrade[All Fields] OR measuretsa[All Fields] OR measureused[All Fields]) OR ("Pract Radiat Oncol"[Journal] OR "pro"[All Fields]) OR ("fetal membranes, premature rupture"[MeSH Terms] OR ("fetal"[All Fields] AND "membranes"[All Fields] AND "premature"[All Fields] AND "rupture"[All Fields]) OR "premature rupture fetal membranes"[All Fields] OR "prom"[All Fields]) |
| **Sensitive search filter** (9) |
| (instrumentation[sh] OR methods[sh] OR Validation Studies[pt] OR Comparative Study[pt] OR “psychometrics”[MeSH] OR psychometr*[tiab] OR clinimetr*[tw] OR clinometr*[tw] OR “outcome assessment (health care)”[MeSH] OR outcome assessment[tiab] OR outcome measure*[tw] OR “observer variation”[MeSH] OR observer variation[tiab] OR “Health Status Indicators”[Mesh] OR “reproducibility of results”[MeSH] OR reproducib*[tiab] OR “discriminant analysis”[MeSH] OR reliab*[tiab] OR unreliab*[tiab] OR valid*[tiab] OR coefficient[tiab] OR homogeneity[tiab] OR homogeneous[tiab] OR “internal consistency”[tiab] OR (cronbach*[tiab] AND (alpha[tiab] OR alphas[tiab])) OR (item[tiab] AND (correlation*[tiab] OR selection*[tiab] OR reduction*[tiab])) OR agreement[tiab] OR precision[tiab] OR imprecision[tiab] OR “precise values”[tiab] OR test–retest[tiab] OR (test[tiab] AND retest[tiab]) OR (reliab*[tiab] AND (test[tiab] OR retest[tiab])) OR stability[tiab] OR interrater[tiab] OR inter-rater[tiab] OR intrarater[tiab] OR intra-rater[tiab] OR intertester[tiab] OR inter-tester[tiab] OR intratester[tiab] OR intra-tester[tiab] OR interobserver[tiab] OR inter-observer[tiab] OR intraobserver[tiab] OR intra-observer[tiab] OR intertechnician[tiab] OR inter-technician[tiab] OR intratechnician[tiab] OR intra-technician[tiab] OR interexaminer[tiab] OR inter-examiner[tiab] OR intraexaminer[tiab] OR intra-examiner[tiab] OR interassay[tiab] OR inter-assay[tiab] OR intraassay[tiab] OR intra-assay[tiab] OR interindividual[tiab] OR inter-individual[tiab] OR intraindividual[tiab] OR intra-individual[tiab] OR interparticipant[tiab] OR inter-participant[tiab] OR intraparticipant[tiab] OR intra-participant[tiab] OR kappa[tiab] OR kappa’s[tiab] OR kappas[tiab] OR repeatab*[tiab] OR ((replicab*[tiab] OR repeated[tiab]) AND (measure[tiab] OR measures[tiab] OR findings[tiab] OR result[tiab] OR results[tiab] OR test[tiab] OR tests[tiab])) OR generaliza*[tiab] OR generalisa*[tiab] OR concordance[tiab] OR (intraclass[tiab] AND correlation*[tiab]) OR discriminative[tiab] OR “known group”[tiab] OR factor analysis[tiab] OR factor analyses[tiab] OR dimension*[tiab] OR subscale*[tiab] OR (multitrait[tiab] AND scaling[tiab] AND (analysis[tiab] OR analyses[tiab])) OR item discriminant[tiab] OR interscale correlation*[tiab] OR error[tiab] OR errors[tiab] OR “individual variability”[tiab] OR (variability[tiab] AND (analysis[tiab] OR values[tiab])) OR (uncertainty[tiab] AND (measurement[tiab] OR measuring[tiab])) OR “standard error of measurement”[tiab] OR sensitiv*[tiab] OR responsive*[tiab] OR ((minimal[tiab] OR minimally[tiab] OR clinical[tiab] OR clinically[tiab]) AND (important[tiab] OR significant[tiab] OR detectable[tiab]) AND (change[tiab] OR difference[tiab])) OR (small*[tiab] AND (real[tiab] OR detectable[tiab]) AND (change[tiab] OR difference[tiab])) OR meaningful change[tiab] OR “ceiling effect”[tiab] OR “floor effect”[tiab] OR “Item response model”[tiab] OR IRT[tiab] OR Rasch[tiab] OR “Differential item functioning”[tiab] OR DIF[tiab] OR “computer adaptive testing”[tiab] OR “item bank”[tiab] OR “cross-cultural equivalence”[tiab]) |
| **Exclusion filter** |
| (“addresses”[Publication Type] OR “biography”[Publication Type] OR “case reports”[Publication Type] OR “comment”[Publication Type] OR “directory”[Publication Type] OR “editorial”[Publication Type] OR “festschrift”[Publication Type] OR “interview”[Publication Type] OR “lectures”[Publication Type] OR “legal cases”[Publication Type] OR “legislation”[Publication Type] OR “letter”[Publication Type] OR “news”[Publication Type] OR “newspaper article”[Publication Type] OR “patient education handout”[Publication Type] OR “popular works”[Publication Type] OR “congresses”[Publication Type] OR “consensus development conference”[Publication Type] OR “consensus development conference, nih”[Publication Type] OR “practice guideline”[Publication Type]) NOT (“animals”[MeSH Terms] NOT “humans”[MeSH Terms]) |
|  |
