# Supplementary material for: Measurement of stiffness in patients with rheumatoid arthritis in low disease activity or remission: a systematic review
Source: BMC Musculoskelet Disord. 2014 Jan 29;15:28. doi: 10.1186/1471-2474-15-28 (PMC3914735; doi:10.1186/1471-2474-15-28)
Supplement: Additional file 2 — Published articles on instruments or subscales of instruments to measure stiffness. [file 1471-2474-15-28-S2.doc]

**Additional file 2:** Published articles on instruments or subscales of instruments to measure stiffness

| **Ref** | **First author + yr of publ** | **Instrument and phrasing** | **Population** |
| --- | --- | --- | --- |
| (25) | Rhind, 1987 | Four stiffness measures:   1. Severity:10 cm VAS from no to very severe 2. Severity: NRS from 0 (no) to 10 (very severe) 3. Severity: 5 point verbal scale with no, mild, moderate, severe and very severe. 4. Duration of MS: ‘*How long did it take for your stiffness to begin to ease after you got out of bed this morning?*’ | 95 RA |
| (31) | Hazes, 1993 | Five stiffness measures:   1. Severity: 10 cm VAS ‘no’ to ‘very severe’ 2. Severity: NRS from 0 ‘no’ to 10 ‘very severe’. 3. Duration of MS: 4. *‘how long does you MS last until it begins to improve?* 5. *‘how long does your MS last until maximum improvement occurs?’* 6. *‘how long does it take you to get going properly?’* | 93 RA + 32 NIC |
| (22) | Hazes, 1994 | Six stiffness measures of duration through a diary and interview;   1. waking to first improvement of MS 2. getting up to first improvement of MS 3. waking to maximum improvement of MS 4. getting up to maximum improvement of MS 5. waking to complete disappearance of MS 6. getting up to complete disappearance of MS | 49 RA |
| (30) | Ward, 1994 | One stiffness measure:  Duration of MS | 24 RA  3 yrs disease duration |
| (6) | Buchbinder, 1995 | One stiffness measure:  Duration of MS in minutes, regarding the day preceding the clinic visit measured as time of awakening to time of cessation. | 142 RA |
| (29) | Vliet Vlieland, 1997 | Two stiffness measures:  Duration of MS:  ‘*how long does your morning stiffness last from waking until maximum improvement occurs?’*  Severity: 10 cm VAS with 0 ‘none’ to 10 ‘very severe’ | 63 +39 trial RA |
| (28) | Sarzi-Puttini, 2002 | One stiffness measure:  Duration of MS | 105 RA, 16 mo disease duration |
| (27) | Yazici, 2004 | One stiffness measure:  Duration of MS in minutes, categorized into 4 groups:  0, 1-15, 16-59, and ≥60 | 337 RA,  18 mo disease duration |
| (10) | Westhoff, 2008 | Two stiffness measures:  Duration of MS  Severity: NRS from 0 ‘ no’ to 10 ‘extremely severe’ | 916 RA patients |
| (32) | Khan, 2009 | One stiffness measure:  Duration of MS min in time from waking to time of max improvement in last week, in 4 categories: none, mild (1-30min), moderate (31-60min) and severe (>60 min) | 5439 RA |
| (19) | Borstlap, 1995 | One stiffness measure:  Severity (?): 10 cm VAS; the lower the score, the more favourable the patients’ condition; no mention of either duration, severity or anchors | 62 OA + 35 RA, 10 yrs disease duration |
| (23) | Houssien, 1997 | One stiffness measure:  Duration of early MS | 200 RA, 11 yrs dd |
| (26) | Wolfe, 1999 | One stiffness measure (WOMAC);  Severity: 2 items in a VAS format, transformed to one score   1. severity at first awakening 2. severity after sitting, lying down or resting during the day | 1013 RA, 625 OA, 531 FM |
| (21) | Fransen, 2000 | One stiffness measure (RADAI)  Duration of MS in 6 categories.  ‘*Were your joints stiff when you woke up today?*  *‘How long did this stiffness last?’ No=o, <30 min=1, 30 min – 1 h=2, 1-2h=3, 2-4h=4, >4h=5, all day=6’* | 584 RA,  8 yrs disease duration |
| (24) | Leeb, 2003 | Two measures of stiffness:  Severity: 2 questions in SACRAH: severity of morning stiffness and daily starting stiffness, from 0 ‘no’ to ‘100’ unbearable.  Duration of early MS in minutes | 103 RA + 69 OA |
| (20) | El Miedany, 2010 | One stiffness measure (PROM)  Duration of MS in minutes from time of awakening.  *‘Over the last week when you awakened in the morning, did you feel stiff? Please indicate the number of minutes, or hours until you are as limber as you will be for the day.’* | 264 RA, 123 PsA + 75 IBD,  4 yrs disease duration |

# RA: rheumatoid arthritis; VAS: visual analogue scale; NRS: numerical rating scale; NIC: noninflammatory conditions; MS: morning stiffness ; FN: fybromialgia ; OA: osteoarthritis; PsA: psoriatic arthritis; IBD: inflammatory bowel disease; WOMAC: Western Ontario and McMaster Universities Arthritis Index; RADAI: rheumatoid arthritis disease activity index; SACRAH: Score for the Assessment and Quantification of Chronic Rheumatoid Affections of the Hands; PROM: multidimensional patient reported outcome measures
